# How oxygen gave rise to eukaryotic sex

**DOI:** 10.1098/rspb.2017.2706

**Published:** 2018-02-07

**Authors:** Elvira Hörandl, Dave Speijer

**Affiliations:** 1 Department of Systematics, Biodiversity and Evolution of Plants, University of Goettingen, Göttingen, Germany; 2 Department of Medical Biochemistry, Academic Medical Centre (AMC), University of Amsterdam, Amsterdam, The Netherlands

**Keywords:** eukaryotes, meiosis, Muller's ratchet, oxidative stress, paradox of sex

## Abstract

How did full meiotic eukaryotic sex evolve and what was the *immediate* advantage allowing it to develop? We propose that the crucial determinant can be found in internal reactive oxygen species (ROS) formation at the start of eukaryotic evolution approximately 2 × 10^9^ years ago. The large amount of ROS coming from a bacterial endosymbiont gave rise to DNA damage and vast increases in host genome mutation rates. Eukaryogenesis and chromosome evolution represent adaptations to oxidative stress. The host, an archaeon, most probably already had repair mechanisms based on DNA pairing and recombination, and possibly some kind of primitive cell fusion mechanism. The detrimental effects of internal ROS formation on host genome integrity set the stage allowing evolution of meiotic sex from these humble beginnings. Basic meiotic mechanisms thus probably evolved in response to endogenous ROS production by the ‘pre-mitochondrion’. This alternative to mitosis is crucial under novel, ROS-producing stress situations, like extensive motility or phagotrophy in heterotrophs and endosymbiontic photosynthesis in autotrophs. In multicellular eukaryotes with a germline–soma differentiation, meiotic sex with diploid–haploid cycles improved efficient purging of deleterious mutations. Constant pressure of endogenous ROS explains the ubiquitous maintenance of meiotic sex in practically all eukaryotic kingdoms. Here, we discuss the relevant observations underpinning this model.

## 1. Introduction

The so-called paradox of sex represents one of the most intriguing problems of evolutionary biology [1,2]. Sex in eukaryotes is a composite process, consisting of meiosis and fertilization (or, more generally, ‘mixis’, the process of fusion of cells and nuclei), which can be coupled to reproduction [3]. Sexual reproduction can be defined as ‘a process in which the genomes of two parents are brought together in a common cytoplasm to produce progeny that may then contain re-assorted portions of the parental genomes' [2, p. 29]. This definition can be relaxed to also include autogamy (self-fertilization), which must be seen as a derived trait, retaining meiosis. Meiosis–mixis cycles are seen as ancestral and conserved features of eukaryotes [4–6].

Altogether, eukaryotic sexual reproduction is a risky, time- and energy-consuming process. Meiosis can break up favourable gene combinations and meiosis itself seems to correlate with higher inviability among potential offspring [7–10]. Recombination at meiosis occurs blindly, chancing new gene combinations in offspring, while recombinant offspring is not necessarily selected for [1]. Mixis entails the cost of a second individual needed for reproduction, with the associated efforts of mate location, conjugation and risks of incompatible mating often leading to inviable or infertile offspring [8,9]. Without sex, a single individual could propagate, avoiding density-dependence of individuals. If only one parent (a ‘female’) is capable of producing offspring, as found in most animals, then asexual females could double their progeny (‘cost of males’, [8]). Again, such considerations do not apply in the case of autogamy.

Numerous hypotheses have been proposed for the maintenance of sex [1,2,11]. Several authors have suggested that the benefits of sex could be found in the repair of damaged DNA [2,12–14], mutation elimination by selection on recombinant offspring [15,16], or restoration of cytosine methylation patterns during meiosis [17]. All these theories offer something, but on their own seem unconvincing, relying on combinational effects and being dominant in certain groups of extant eukaryotes only [18]. Here, we try to reconstruct a likely evolutionary order of events, taking into account physiological and biochemical constraints of eukaryotic life.

Repair of chemically altered DNA offers itself as the primary force, as it constitutes an immediate cellular constraint; transcription and replication cannot proceed with chemically damaged DNA [13]. DNA damage is mostly caused by reactive oxygen species (ROS) and includes modification by oxidation, resulting in single- and double-strand breaks (DSBs), formation of DNA adducts and cross-links [19]. Crucially, single ROS initiation events can generate multiple reactions and radical molecules by complex chain reactions (mostly catalysed by metal cations in Fenton reactions) that affect all cell components [20]. ROS, except for H_2_O_2_, have extremely short half-lives. However, they almost always initiate chain reactions of cell (even tissue-wide) oxidations, the specificities of which depend on the chemical environment [21]. The absence of DNA repair can be lethal immediately, whereas an incorrect base repair will lead to mutations (stable changes in the sequence of DNA base pairs [22]). Mutations can efficiently be eliminated or favoured by Darwinian selection (genetic drift being ‘blind’). A tiny fraction of mutations turn out to be positive, but most are neutral or negative, ranging from mildly disadvantageous to deleterious. Selection against accumulation of deleterious mutations is most efficient among recombinant offspring [15]. However, this is not an immediate, but a more long-term effect, strongly modulated by group-size, severity and epistatic interactions of mutations [16].

We postulate that initial endogenous ROS formation by the endosymbiont and resulting DNA damage in early stages of eukaryogenesis could have triggered meiosis–mixis cycles. Subsequently, high-energy metabolism (involving respiration and photosynthesis) and developments based on adaptations to effects of endogenous ROS production were among forces giving rise to (complex) multicellularity with germline/soma differentiation. At this stage, elimination of mutations by purifying selection added a major advantage of sex for multicellular, long-lived, diplontic or diplohaplontic life cycles [23]. Eventually, meiotic resetting of DNA methylations became important, especially for complex multicellular metazoans. At this point, we should stress that parts of this ‘ROS-sex’ hypothesis are much debated and not yet (?) universally accepted.

## 2. Did endogenous oxidative stress trigger sex at the origin of the eukaryotes?

How did eukaryotes end up burdened with meiosis–mixis cycles? Considering genetic variability and adaptive potential, prokaryotic forms of gene exchange (plasmid-mediated conjugation, phage-mediated transduction and transformation [24]) have allowed an enormous quantity of hugely diverse organisms to evolve. Prokaryotes are highly adaptive, exhibit numerous trophic forms and have colonized a tremendous variety of habitats on our planet. Having network-like ‘pangenomes’, prokaryotes can transfer genetic material from one individual to another, unrestricted by meiosis–mixis cycles, resulting in countless gene combinations [25]. Selection can act efficiently on the huge genetic diversity present, owing to large prokaryotic population sizes. This results in survival and adaptation of strains to novel environments, as illustrated by rapid evolution of antibiotic resistance in pathogenic bacteria. To maintain genetic variability, a meiotic process seems superfluous in this case.

But with eukaryotes, the rules of the game change. They are mostly limited to vertical inheritance with gene exchange restricted to genetically very similar individuals [25]. Wilkins & Holliday [26] suggested that meiosis, in fact, may have evolved to *restrict* recombination events rather than promote them, and Bernstein *et al.* [12] were among the first to show that most meiosis events do *not* result in recombination. Also, mutation accumulation (think ‘Muller's ratchet’) does not seem a strong argument for *starting* with meiosis—prokaryotes have their own antioxidant defences (see, e.g. [27] and, even more importantly, detrimental prokaryotic mutations are effectively purged from large populations). However, in eukaryotes, Muller's ratchet is a much bigger problem (see below).

The origin of sex might have been the appearance of meiosis as a superior nuclear DNA repair mechanism in the wake of rising oxygen levels in the Earth's atmosphere in the Proterozoic, caused by oxygenic cyanobacterial photosynthesis. Oxygenic photosynthesis evolved earlier, in the Archaean, with several markers first appearing approximately 2.5 × 10^9^ years ago [28]. Oxygenic photosynthetic organisms use light energy for photochemical oxidation of water, releasing oxygen, to generate chemical energy (ATP) and reduction equivalents (NADPH). Both are required to synthesize carbohydrates in the Calvin cycle, beginning with CO_2_ fixation, catalysed by Rubisco. Oxygen, the waste product of photosynthesis, thus became enriched in the atmosphere and in bodies of water [29]. Some heterotrophic alpha-proteobacteria managed to link the breakdown of organic matter to short-chained organic acids with their further oxidation by aerobic respiration, giving CO_2_ and water as waste, while the energy thus gained is stored as ATP. Both photosynthesis and respiration involve complex electron-transfer chains that secure the transfer of four electrons. Accidental one-electron transfers generate highly ROS in intermediate steps of the chemical reactions [20], simplified as follows:

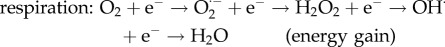


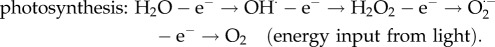


The different ROS are: 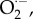 superoxide radical; H_2_O_2_, hydrogen peroxide; OH^·^, hydroxyl radical.

For overviews of the reactions in ROS and reactive nitrogen species chemistry, see [30] and chapter 6 in [31].

**Figure 1.**
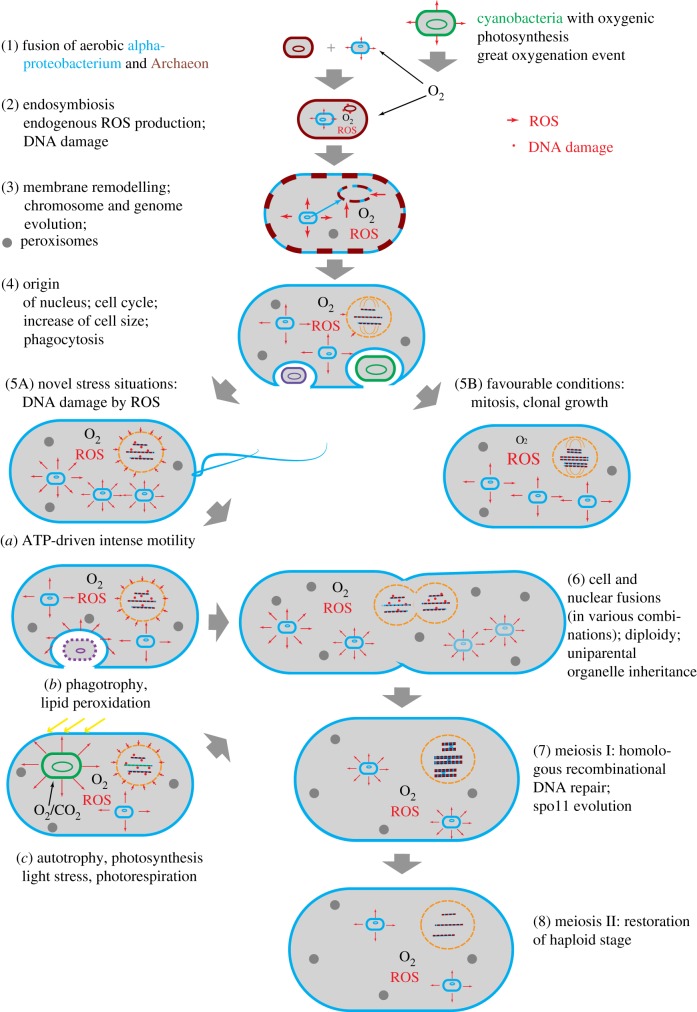
Possible steps describing eukaryotic origins and evolution of meiotic sex. The specific timing is arbitrary (e.g. meiotic sex probably evolved before phagocytosis). (1) Cell fusion of Archaeon and alpha-proteobacterium; (2) establishment of endosymbiosis with aerobic respiration, efficient energy generation and internal ROS production; (3) remodelling of membranes, origin of peroxisomes, transition to linear host chromosomes, chromatin, transfer of genes from mitochondrial genome to host genome and RNA splicing; (4) endogenous evolution of nuclear envelope for protection from short-lived ROS, spindle formation for moving bulky linear chromosomes, establishment of mitosis, mitochondrial ATP production allowing increase of body size (and phagocytosis?); (5A) novel stress situations with ROS (H_2_O_2_) production and increase in nuclear DNA damage: e.g. high motility, phagotrophy, endosymbiosis with cyanobacteria (the first plastid acquisition is difficult to date, but probably earlier than previously thought, [33,34]); (5B) mitosis and clonal growth as an alternative mode of reproduction under favourable conditions; (6) DNA damage triggers cell and nuclear fusions in various combinations, leading to early eukaryotic, mostly mixotrophic, panmictic (?) populations; (7) meiosis I established as HR DNA repair tool, homologous pairing established by controlled DSB formation, lineage-specific spo11 evolution; and (8) meiosis II and establishment of diploid–haploid cycles. (Online version in colour.)

In our view, eukaryogenesis started when an Archaean host (or merging Archaeons; as hypothesized in [32]) established endosymbiosis with free living, (facultatively) aerobic alpha-proteobacterium-like organisms which became mitochondria in an example of syntrophy (figure 1). How uptake took place is unclear. We will not discuss proposed mechanisms, but we consider primitive forms of phagocytosis unlikely [35]. Later, endosymbiosis with photosynthetic cyanobacteria resulted in plastids [25,36–38].

The first eukaryotes benefitted from the high-energy gain of aerobic metabolism (and the occasional photosynthesis), allowing further cellular complexity and larger size, but suffered from severely increased *internal* ROS production [6,38,39]. Transcription and replication open double-stranded DNA, with single strands coming under oxidative attack, potentially causing breaks and other lesions [22,40]. We contend that meiotic sex evolved in the context of endogenous ROS production by the pre-mitochondrion, with homologous DNA repair as its initial starting point. Other (not mutually exclusive) models also trace the origin of meiotic sex to the first endosymbiont appearance (cf. [6,41,42]). Another important ‘ROS response’ could have been nuclear membrane development, surrounding the host genome, made from secreted endosymbiont vesicles [43]. The availability of abundant ATP from oxidative respiration for membrane formation is another argument for the endogenous origin of a nuclear envelope [42]. The nuclear envelope might have protected the host genome from oxidative stress because of organelle-derived ROS (see also [44–46]). Endogenous ROS probably also contributed to the evolution of a eukaryotic cell cycle: unlike in prokaryotes, transcription, DNA synthesis and cell division proceed in separated phases (though Archaea, interestingly, are more ordered in this respect [47]). While not central to the ‘ROS-sex’ hypothesis, others have proposed further benefits or consequences of nuclear membrane formation. For instance, the nucleus also allowed mRNA splicing before export to the cytosol and translation [48]. Endosymbiosis further led to transfer of many organellar genes to host genomes [25,49,50] and the development of new organelles such as peroxisomes (see below). A further large increase of host genome DNA length was owing to concomitant intron integration [48]. This expansion of the host's genome may have been a factor in the appearance of linear chromosome structures, which can maintain larger (repetitive) genomes more efficiently than ring-like ones [51].

Further innovations leading to mitosis and meiosis were the organization of DNA in chromatin, in linear chromosomes with kinetochores and a division mechanism with a spindle apparatus. The long eukaryotic DNA strands are densely packed in nucleosomes, i.e. DNA is wrapped around histones. Strikingly, Archaea possess histones [52]. This predisposition allows that, with only a few steps for the concerted evolution of chromosome condensation, nucleosome and centromere formation, eukaryotic chromosome structures could have evolved [53]. In the first eukaryotic cell divisions, nuclear membrane components may have helped to separate chromosomes, while the nuclear spindle emerged at later stages [32,54]. Eukaryotic chromatin became bulky, and a more efficient mechanism using microtubules was needed to pull chromatids apart. Microtubuli have the same structure as eukaryotic flagellae (‘undulipodia’ *sensu*, [3]) which simply points at the evolution of a general, robust, tear-resistant mechanical structure in early eukaryotes. Tubulin homologues and SMC structural maintenance of chromosome proteins had already evolved in prokaryotes [26]. The mitochondrion provided the copious amounts of ATP required for microtubule and nuclear spindle formation [42]. As we argue that meiotic sex originated and ‘matured’ in the context of internal pre-mitochondrial ROS formation, coordinated cell/organelle division should be ancient. Organellar inheritance in eukaryotes is dominated by uniparental organelle inheritance (UPI). A detailed hypothetical evolutionary scenario resulting in UPI is given in the electronic supplementary material, S1. Organellar DNA is protected from permanent ROS damage by specific antioxidant enzymes such as superoxide dismutase and glutathione transferase [55], by using gene conversion as a DNA repair mechanism [56,57] and by transfer of many genes to the nucleus (see above), while genes encoding some of the hydrophobic core subunits of the large membrane complexes are kept, possibly to maintain redox control [58,59]. Moreover, purging selection can act on large populations of organelles inside most modern eukaryotic cells and remove malfunctioning ones.

## 3. Sources of endogenous oxidative stress in heterotrophic and autotrophic eukaryotes

Meiosis and mitosis probably evolved concurrently in early eukaryotes [4]. Mitosis probably was the main process for clonal reproduction under favourable conditions, while meiosis represented an occasional modification of mitosis acting under DNA-damaging stress conditions (figure 1). Correlation of meiosis with oxidative stress was demonstrated in many extant eukaryotic groups exhibiting facultative sexuality/asexuality [60–64]. Upon increased competition between eukaryotes, tiny innovations to obtain food were positively selected, culminating in full-scale phagocytosis. Larger body size and the availability of mitochondrion-derived ATP allowed many eukaryotic lineages to become phagocytotic later on [35]. However, phagocytosis might renew physiological stress. Additional ROS could, for example, have arisen from extraordinary high mobility, when mitochondrial ATP production had to be rapidly intensified to allow intense flagellar movement, in competition for organic molecules (or escaping an adverse environment). Interestingly, incomplete non-digestive phagocytosis of cyanobacteria might have led to photosynthetic (i.e. autotrophic) eukaryotes. Photosynthesis has its own sources of, surprisingly high, endogenous oxidative stress (an extensive overview of which is given in the electronic supplementary material, S2), which explains the need of meiotic sex in autotrophic (or mixotrophic) eukaryotes.

A further potential source of ROS production in phagotrophic eukaryotes was food rich in proteins and very long saturated fatty acids, the main component of membranes, requiring breakdown by β-oxidation. In mitochondria, the respiratory chain seems optimized to use glucose as a substrate; β-oxidation would be energy-efficient but leads to ROS formation [65]. This aspect (β-oxidation, occurring *prior* to phagocytosis) probably triggered the evolution of novel organelles, peroxisomes, performing β-oxidation without concomitant mitochondrial ROS formation. The H_2_O_2_ generated instead is efficiently scavenged inside the organelle by catalase [65]. Recently, it was found that in human fibroblasts without peroxisomes, peroxisomal import receptors Pex3 and Pex14 go to mitochondria and are subsequently released in pre-peroxisomal vesicles (again stressing their postulated evolutionary link). These vesicles fuse with Pex16 containing endoplasmatic reticulum (ER)-derived vesicles, giving rise to peroxisomes (defined as vesicles capable of import of peroxisomal proteins) [66]. Whether peroxisomes evolved prior to the ER, or concomitantly, is not clear. We previously described peroxisome evolution in the context of phagocytosis, but peroxisomes (and ER?) probably evolved earlier: directly on the heels of the uptake of the pre-mitochondrion and the integration of host and endosymbiont metabolic pathways [67], as illustrated by the many ‘new’ transporter systems they share with mitochondria [68].

## 4. Evolution of meiosis as a response to oxidative damage

H_2_O_2_ can arise from many eukaryotic metabolic processes, easily penetrates (nuclear) membranes and reacts in the presence of transition metals (especially iron), via so-called Fenton chemistry, to produce extremely aggressive hydroxyl radicals [20]. Eukaryotic nuclei, especially nucleoli, have high iron concentrations [69]. Hydroxyl radicals are especially dangerous for DNA as they can lead to tandem lesions [70]. Thus, cells need to react with, for example, oxidatively damaged peptides functioning as secondary ROS messengers entering the nucleus [71]. One could speculate that in early phases of eukaryotic evolution, ROS-induced DSBs of the host's DNA and incomplete repair of these breaks could have caused the transition from a single Archaeal ring-like chromosome to several shorter, linear chromosomes, characteristic of eukaryotes. Linear ends have to be protected against exonuclease activity, necessitating telomere restoration to allow full replication. As proposed by Garavis *et al*. [72], early eukaryotes could have ‘solved’ this end replication problem using G-quadruplexes and retrotransposon activity. Moreover, linear chromosome structures can be better aligned for precise homologous recombinational (HR) repair than ring-like chromosomes [51], fitting in with more efficient DNA repair mechanisms coping with ROS-produced damage upon the merger leading to the eukaryotic lineage.

The main novelty of meiosis compared to mitosis is homologue pairing and synapsis at prophase I [26], and here HR repair of DSBs during meiosis occurs [73]. Homologous recombinational repair using a second, homologous DNA molecule is the most accurate and least mutagenic DNA repair mechanism [22]. Cell fusion could have provided a mechanism in early eukaryotic evolution to get this second homologous DNA molecule. By merging and combining nuclear genomes, similar chromosomal structures could align, and HR repair established in the eukaryotic zygote. Indeed, Archaea were probably already capable of cell fusion [42]. Whether it occurred regularly or not, one might speculate that loss of archaeal electron transport chains and their associated membrane potentials in evolving eukaryotes [38] made it easier. Archaea thus might have been predisposed to mixis. Archaeal DSBs can be induced by exogenous oxidative stress caused by ultraviolet light or chemicals [32]. Furthermore, all Archaean DNA repair proteins required for HR repair of DSBs were available, forming the homologues of core meiotic proteins [5,74]. Only proteins for chromosomal homology search and binding seem absent [5]. But, homology search and synapsis must have rapidly been established (selection acts strongly against recombinational errors caused by non-homology [26]). Even the endosymbiont could have contributed to homology searching, as stress-induced genome condensation leads to non-random convergence of sister chromosomes culminating in spatial proximity of homologous sites in bacteria [75]. If internal ROS creates an environment in which tiny steps towards meiotic sex are selected for, why did it not evolve in the mitochondria themselves? The specific archaeal ‘predispositions’ possibly explain this, and large-scale migration of endosymbiont genes to the protected nuclear environment quickly started to function as an efficient alternative protection against ROS-induced damage.

This first HR repair in zygotes was probably the precursor of prophase I of meiosis. Indeed, in all extant eukaryotes studied so far, prophase I is the most conserved and least dispensable step in various forms of meiosis modifications in different modes of reproduction [76]. In contrast with bacterial transformation, meiosis-like repair was reciprocal, as both fusing individuals had an immediate selective advantage: rescuing their genomes [51]. Hence, mixing cells had just to combine pre-existing mechanisms of Archaea, i.e. cell fusion and existing HR DNA repair tools, to repair chromosomes. Further steps of meiosis mostly just represent modifications of mitosis: alignment of chromosomes in metaphase I; separation of homologous chromosomes at anaphase I without centromere splitting and absence of sister chromatid separation, possibly causally linked to suppression of the synthesis-phase after meiosis I [26]. Meiosis II is just a mitosis and regenerates haploidy, and this way the first meiosis–mixis cycles could have been established. The regular establishment of diploid–haploid cycles probably happened later, mostly in multicellular eukaryotes. The diversity of meiosis variants in protists supports the hypothesis of a stepwise establishment process with many experimentations [77]. Meiotic recombination with its typical extant features (e.g. synaptonemal complexes) probably evolved *after* establishment of meiosis–mixis cycles [26].

Looking at the evolution of mitosis and meiosis–mixis cycles retrospectively (figure 1), it might seem surprising that so many novel processes and structures were combined, and that intermediate forms are largely missing. However, by combining *complete* genomes (initially paired just for HR DNA repair), and by introducing *reciprocal* recombination events, sex could rapidly exchange and fix the *gene combinations* that encode proteins for all kinds of ‘new’ eukaryotic features (e.g. nuclear envelope, cell cycle, mitosis and meiosis) in the offspring. Hence, the successful *combination of features* could spread much faster in sexual populations than any single innovation that might have appeared in mitotic lineages. With meiotic sex, eukaryotes gave up rapidly producing novel genotypes the way prokaryotes do (less new features coming from horizontal transfer), but they gained a reproductive system that allowed efficient generation and vertical inheritance of powerful combinations of molecular features.

## 5. Sex, multicellularity and evolution of complex organisms

Many unicellular eukaryotes can persist without meiotic sex over very long periods, though real clonality seems to be extremely rare even in single-celled organisms [3,6]. Even pathogenic microbial eukaryotes require sex as a genomic repair tool upon encountering the host's defence [78]. Unicellular eukaryotes face the problem that meiosis is a time and energy-consuming process, lasting several hours in which other cellular activities have to be put on hold. Moreover, having just one nucleus means that an erroneous meiosis probably is lethal for offspring. The first problem is sometimes met by differentiating two nuclei, one vegetative macronucleus for protein–transcription and cell functions and one generative micronucleus for meiosis and reproduction (e.g. in extant ciliates like *Tetrahymena*, [79]). Mixis, as the second component of sex, requires reachable mating partners with homologous genomes, but small organisms cannot move far. Small body size could make sex costly [9]. With regard to multicellular organisms, many arguments rather speak for a regular use of meiotic sex: (i) the fossil record; (ii) the advantages of sex for multicellular development starting from single-cell stages; (iii) the advantages of a germline–soma differentiation, such as allowing multicellular organisms to restrict ROS-producing functions as much as possible to somatic cells (e.g. [58,80] and references therein; electronic supplementary material, S3).

The oldest multicellular fossil with morphological structures indicative of sexual reproduction is the red algae-like *Bangiomorpha pubescens* [81]. This organism developed multicellularity from single-cell stages via mitotic divisions before forming structures for sex [81]. Multicellular life forms evolved many times, and multicellularity is not restricted to eukaryotes [82]. Multicellularity provides many advantages, e.g. protection against predation, efficient food consumption, facilitating dispersal and division of labour among cells. Simple cellular colonies start with benefits from an increased buffering of physical and biological environmental influences, and from intercellular metabolic exchange [83]. However, multicellular prokaryotes lack central developmental programmes, and thus remain without significant cell differentiation [82]. Complex multicellular eukaryotes differentiate an immortal germline from a mortal somatic line [18]. Only the germline needs meiotic repair (see details in the electronic supplementary material, S3).

Although early eukaryotes managed to keep ROS production under control with various mechanisms, they could not scavenge ROS completely, a feat nearly impossible to accomplish [27]. Making a virtue of necessity, ROS emission probably was used early on for signalling from the organelle to the nucleus in the service of metabolic adaptations. Later on, positive effects in cell differentiation, as well as in stress responses, such as encountered upon microbial pathogen attack turned out to be valuable [20,21,84,85]. The danger of intra-nucleate oxidative damage of DNA persisted, but probably rather in the formation of local DNA damage than in causing direct DSBs, the former being much more frequent than the latter [22]. Hence, prophase I of meiosis could have been optimized for conducting HR repair of the more frequent minor lesions (e.g. owing to local DNA radicals) in germline cells [86]. Certain spo11 orthologues (which probably evolved earlier as a radical-scavenging enzyme in Archaea) induce meiosis-specific DSBs in all eukaryotic kingdoms [73]; in protists, e.g*.* in the ciliate *Tetrahymena* [79]. Spo11 action results in a controlled DSB formation which is afterwards repaired [73,86]. In most extant multicellular eukaryotes, a minimum of one spo11-induced DSB is needed to initiate meiosis and to guarantee correct segregation [87]. Meiotic DSB breaks do not occur randomly, but in hotspots; in mice, they are mostly found in between methylated nucleosomes [88]. Maybe these regions are less protected against oxidative damage (in line with the idea that eukaryotic chromosomal structures came about because of internal ROS pressure)? Whatever the truth of this supposition, many more DSBs are made than are later on repaired via a crossing-over pathway, which speaks in favour of the repair function rather than for a teleological ‘purpose’ of recombination [86]. This costly HR DNA repair is primarily reserved for immortal germline cells, while accumulation of oxidative damage and mutations derived from non-HR repair in somatic cells is an important factor in ageing and death [89] (see also the electronic supplementary material, S3). A rare exception are asexual bdelloid rotifers which exist for millions of years without meiotic sex by using extraordinary efficient antioxidant systems and gene conversion to eliminate mutations [90].

DNA repair happens at meiosis I, but it cannot explain meiosis II and reductional division. Here, heritable mutations as an indirect consequence of oxidative stress come into play [18]. Mutation accumulation does not play a major role during prokaryotic evolution—defective mutants are rapidly purged by selection, and slightly deleterious mutations can never start to dominate the population as effective bacterial population size is large. However, Muller's ratchet depends on mutation rate and genome size, both increasing dramatically upon the merger that gave rise to the eukaryotes, as well as on effective population size, (strongly) decreasing in eukaryotes (as is to be expected, based on their higher energy needs). These problems (more damage, larger genomes and small populations) increase even further in complex multicellular organisms with prolonged lifespans. Mutations can accumulate over generations: first, mutations in germline cells would not immediately affect the viability of the whole parental organism; second, in diploid or polyploid nuclei, i.e. in zygotes, recessive deleterious mutations can remain masked by unmutated gene copies protecting the mutation from purging selection (i.e. heterosis) [23,91,92]. Complex multicellular organisms are diplontic or diplohaplontic (animals and vascular plants, respectively) and do their somatic differentiation in the ‘buffered’ diplo-phase. Diploidy (and polyploidy) can be a result of mixis. However, in the long run, a continued increase of genome size by continuing cell fusions is problematic: space in the nucleus and resources for synthesis of larger amounts of DNA are limiting factors [93]. Moreover, outcrossing via haploid gametes promotes heterosis as a beneficial effect. In the light of these considerations, reductional divisions are favoured by selection to reduce ploidy levels.

Theoretically, meiosis is an efficient mutation purging mechanism of ‘masked’ deleterious mutations owing to the return to a haploid phase in gametes, because selection acts more efficiently on haploids [94]; in multicellular organisms, selection can act on the haploid, recombined products of meiosis (gametes or in plants, gametophytes) and eliminate mutants [18,95–97]. Theoretical models revealed that surprisingly little recombination resulting from facultative sexuality is sufficient to counteract mutation accumulation [98]. Gene conversion, the more frequent product of prophase I, is even more efficient as mutations become homozygous and fully exposed to purging selection [90,99]. Gene conversion might also prevent mutation accumulation in non-recombining genomes like plastids and mitochondria [56,57].

In multicellular, differentiated, organisms, the DNA restoration mechanism of resetting cytosine methylation status during meiosis [17] came into play. In animals and plants, DNA methylation regulates epigenetic silencing of gene expression and control of transposable elements, and hence is important for tissue differentiation [100]. The detailed mechanisms of meiotic resetting and transgenerational inheritance of methylations are complex and differ between plants and animals [101]; it would be outside the scope of this paper to treat this topic in detail. We just mention one point here: that meiotic resetting of methylation profiles makes sense for germ line cells and cells undergoing differentiation, but not for differentiated somatic cells that have lost their totipotency during development.

## 6. Some remarks on Darwinian evolution and conclusion

One of the many observations strongly supporting Darwin's evolutionary model is the strange mixture of adaptive and seemingly useless features of organisms we find in abundance. These reflect historical contingencies that earlier traits, once selected for, but now hampering optimality, represent. Here, we can encounter quite a few examples, operating at different levels. We think that it worthwhile to mention just two: (i) cyanobacteria produced large amounts of oxygen via photosynthesis, irreversibly changing the environment. Their descendants, chloroplasts, do so *inside* the cell, raising O_2_/CO_2_ ratios. The resulting photorespiration (Rubisco-binding O_2_) produces ROS and wastes energy; and (ii) we think that internal ROS formation and DNA damage gave rise to ‘expensive’ meiotic sex, which organisms tend to discard only under certain circumstances when the meiosis–mixis cycle is disrupted, e.g. after hybridization or polyploidization [18]. How many of the independently evolved clonal lineages are stable over longer timescales remains to be seen [2,6].

The dynamic process of having to adapt to the constantly changing environment resulting from other organisms adapting makes evolutionary reconstruction both very exciting and very challenging. We think that meiotic sex is ‘a consequence of oxygen’, because there are many indications that it started out as a repair mechanism for internal, constant ROS-induced DNA damage and elimination of heritable mutations, along the lines we sketched, but realize that many in the field are not convinced, precisely because it is so deeply buried under layers of later adaptations. We show that the physiology of eukaryotes caused novel, ROS-producing stress situations which made a highly efficient DNA repair mechanism indispensable.

With the combined advantages of all restoration mechanisms, the large majority of all eukaryotes maintained meiosis–mixis cycles. In the evolutionary order of events, repair of oxidative damage was the first step as a response to endogenous ROS production by mitochondria, and later on, by plastids, and this happens during prophase I of meiosis. Indeed, prophase I of meiosis is the most indispensable phase of sex [79]. Its repair function is indispensable because of oxidative respiration, and later on, photosynthesis. Endogenous ROS production became intertwined with complex multicellularity and cell differentiation, and in multicellular organisms, sex became thus even more important for selective elimination of mutations and perhaps for resetting of DNA methylation patterns. The selective advantages of having high-energy metabolisms (oxidative respiration and water-dependent photosynthesis) combined with multicellular tissue differentiation require meiotic sex for maintaining the integrity of the immortal germline. At every conceivable level, ROS thus have had an enormous influence during eukaryotic evolution.

Future research should focus on phylogenomic reconstructions of evolutionary history, physiology and reproductive features of early eukaryotes. Experimental and biochemical work with extant unicellular eukaryotes and asexual organisms will help in understanding different functions of the components of sex. Mathematical modelling needs to consider regulatory complexity and the ubiquitous selective pressure of oxidative damage. Sex cannot be understood with short-term cost–gain calculations in extant organisms without considering long-term evolutionary histories.

## Supplementary Material

The evolution of patterns of organelle inheritance in relation to the evolution of meiotic sex.

## Supplementary Material

Sources of endogenous oxidative stress in photosynthetic eukaryotes

## Supplementary Material

The interplay of multicellularity, meiotic sex and ROS

## Data accessibility

The data is supplied in three supplementary files in the electronic supplementary material.
