# Supplementary material for: How oxygen gave rise to eukaryotic sex
Source: Proc Biol Sci. 2018 Feb 7;285(1872):20172706. doi: 10.1098/rspb.2017.2706 (PMC5829205; doi:10.1098/rspb.2017.2706)
Supplement: The evolution of patterns of organelle inheritance in relation to the evolution of meiotic sex. [file rspb20172706supp1.docx]

**Supplementary electronic materials S1:**

**The evolution of patterns of organelle inheritance in relation to the evolution of meiotic sex.**

We argue that meiotic sex originated and "matured" in the context of internal ROS formation by the pre-mitochondrion. Thus, coordinated cell/organelle division should be an ancient trait. Overall, a rather unsophisticated "stochastic" mechanism can be envisaged first, consistent with the idea that sex evolved in the context of endosymbiont uptake with relatively few organelles. Indeed, we suggest that early eukaryotes had only few organelles, as in some present-day unicellular eukaryotes, followed by a general increase and variations in number of organelles, as we now can observe in multicellular eukaryotes (see [1]). Possibly, a mostly random distribution of organelles over the daughter cells would give selective advantage to organelles that *“overreplicate”.* This, in turn, would lead to an overabundance of organelles and the necessity of "organelle downregulation" upon cellular fusion. This might help us understand uniparental organelle inheritance (UPI). Thinking along the lines of Bendich, we propose that UPI is a “…incidental consequence of the demise of organellar DNA, which provides some biochemical benefit.” [2], the benefit being a reduction of *overcapacity* organelles (of course including expensive organellar DNA). A simple mitophagic mechanism, if already available, might have been unreliable with regard to the number of organelles left. Coupling this basic reduction mechanism to a pathway only targeting organelles coming from one parental source (and this *later* development indeed uses many quite different mechanisms), would automatically give an appropriate number. This could explain the existence of isogamous protists with strict uniparental inheritance. Later adaptations could be manifold: e.g. specifically ROS-derived DNA damage [3]; see also [4] and references therein. Our reconstruction does not exclude proposals that UPI also increases the efficiency of purifying selection, but this might not have been the "original driving force". Many, often non-mutually exclusive, explanations for the existence of UPI have been proposed. A concise and nicely written overview of the extent of UPI and the problems associated with understanding it can be found in [5].

**References**

1. Cole LW. 2016 The Evolution of Per-cell Organelle Number. *Frontiers in cell and developmental biology* **4**, 85-85. (doi:10.3389/fcell.2016.00085).

2. Bendich AJ. 2013 DNA abandonment and the mechanisms of uniparental inheritance of mitochondria and chloroplasts. *Chromosome Res.* **21**(3), 287-296. (doi:10.1007/s10577-013-9349-9).

3. Allen JF. 1996 Separate sexes and the mitochondrial theory of ageing. *J. Theor. Biol.* **180**(2), 135-140. (doi:10.1006/jtbi.1996.0089).

4. Speijer D. 2016 What can we infer about the origin of sex in early eukaryotes? *Phil. Trans. Royal Society B-Biol.Sci.s* **371**(1706). (doi:10.1098/rstb.2015.0530).

5. Birky CW, Jr. 2008 Uniparental inheritance of organelle genes. *Curr. Biol* **18**(16), R692-695. (doi:10.1016/j.cub.2008.06.049).
