# Supplementary material for: How oxygen gave rise to eukaryotic sex
Source: Proc Biol Sci. 2018 Feb 7;285(1872):20172706. doi: 10.1098/rspb.2017.2706 (PMC5829205; doi:10.1098/rspb.2017.2706)
Supplement: Sources of endogenous oxidative stress in photosynthetic eukaryotes [file rspb20172706supp2.docx]

**Supplementary electronic materials S2:**

**Sources of endogenous oxidative stress in photosynthetic eukaryotes**

Interestingly, the plastids could have been the first endosymbionts, which, in contrast with the earlier mitochondrial scenario, were due to incomplete non-digestive phagocytosis of cyanobacteria. Their later uptake gave rise to autotrophic eukaryotes. However, just as with mitochondria, not a trace of a “phagocytotic” membrane is left, so this uptake might also have occurred before the emergence of phagocytosis. A recent publication suggests the cyanobacterial lineage diverging from the plastid lineage 2.1 x 10^9^ years ago; while the ancestor of photoautotrophic eukaryotes possibly lived 1.9 x 10^9^ years ago [1]. Thus, photosynthetic eukaryotes could be among the oldest eukaryotic clades. However, basal nodes of the eukaryotic tree are not well resolved and lack statistical support [2, 3]. Complicating matters, some key meiosis genes already duplicated in LECA, have separate histories of duplication/loss in plant and protist lineages as compared to the animal/fungus clade [4].

Regardless of the timing of events, photosynthesis is the major source of oxidative stress in photoautotrophic organisms which do not suffer so much from stress factors of heterotrophic metabolism (intense motility, phagotrophy), but nevertheless do have meiotic sex. Modern plant cells have 30-100 times higher H_2_O_2_ levels than animal cells [5]. In photosynthetic (or mixotrophic) eukaryotes, exposure to high light intensities or prolonged photoperiods as well as deceleration of the Calvin cycle when CO_2_ is limited, create transferable electrons in abundancies conductive to ROS production [6]. Basically, if more electrons are available than receptor molecules other than oxygen, ROS as superoxide anion radical and H_2_O_2_ can arise. Higher relaxation of photosynthetic pigments due to high light amounts results in reactive singlet oxygen which is reduced more easily to superoxide anion radicals than triplet oxygen [7, 8]. Mirroring alpha-proteobacteria (in the form of mitochondria), endosymbiontic cyanobacteria (in the form of plastids), now produce this inside eukaryotic cells, instead of releasing it into the surroundings. Photorespiration acts as a further internal ROS source, in the form of H_2_O_2_. This is a light-induced, temperature-sensitive process, in which Rubisco uses oxygen instead of CO_2_ as electron acceptor, depending on their relative concentrations. The resulting 2-phosphoglycolate can be metabolized in peroxisomes via a sequence of energy-consuming reactions, during which H_2_O_2_ is released. Photorespiration is a disadvantageous side-process of photosynthesis as it reduces both net carbon fixation and growth [9]. Photosynthesis and CO_2_ fixation via Rubisco evolved in the early earth’s atmosphere, in which oxygen concentrations were too low to compete with CO_2_, and oxygen produced by cyanobacteria was released in the surrounding water. But, as endosymbionts, chloroplasts release oxygen inside the cell, raising relative concentrations of oxygen versus CO_2_. With the increase of intracellular oxygen concentrations, photorespiration became a significant ROS producing and energy-consuming competitive pathway in plant metabolism; only some of the most recent lineages of flowering plants are able to reduce photorespiration efficiently via C4 photosynthesis [9]. Thus, also photorespiration exemplifies the oxygen paradox of life: oxygen allows very efficient energy (ATP) generation, but has severely detrimental effects because it leads to ROS induced molecular damage [10, 11]. In the dark, most oxygen radicals in plants are produced by mitochondria (as in heterotrophic organisms) [12]. In both mitochondria and plastids, separate genomes were retained, possibly because the genes encode proteins which allow direct responses to changes in redox states under different environmental conditions [13].

**References**

1. Sanchez-Baracaldo P, Raven JA, Pisani D, Knoll AH. 2017 Early photosynthetic eukaryotes inhabited low-salinity habitats. *Proc. Natl. Acad. Sci.* **114**(37), E7737-E7745. (doi:10.1073/pnas.1620089114).

2. He D, Fiz-Palacios O, Fu C-J, Fehling J, Tsai C-C, Baldauf Sandra L. 2014 An Alternative Root for the Eukaryote Tree of Life. *Curr. Biol.* **24**(4), 465-470. (doi:<http://dx.doi.org/10.1016/j.cub.2014.01.036>).

3. Pawlowski J. 2013 The new micro-kingdoms of eukaryotes. *BMC Biol.* **11**, 3. (doi:10.1186/1741-7007-11-40).

4. Malik SB, Ramesh MA, Hulstrand AM, Lodgson JM. 2007 Protist homologs of the meiotic *Spo11* gene and topoisomerase VI reveal an evolutionary history of gene duplication and lineage-specific loss. *Mol. Biol. Evol.* **24**(12), 2827-2841. (doi:10.1093/molbev/msm217).

5. Hossain MA, Bhattacharjee S, Armin S-M, Qian P, Xin W, Li H-Y, Burritt DJ, Fujita M, Tran L-SP. 2015 Hydrogen peroxide priming modulates abiotic oxidative stress tolerance: insights from ROS detoxification and scavenging. *Front. Plant Sci.* **6**, 420. (doi:10.3389/fpls.2015.00420).

6. Rinalducci S, Murgiano L, Zolla L. 2008 Redox proteomics: basic principles and future perspectives for the detection of protein oxidation in plants. *J. Exper. Botany* **59**(14), 3781-3801. (doi:10.1093/jxb/ern252).

7. Foyer CH, Noctor G. 2009 Redox regulation in photosynthetic organisms: signaling, acclimation, and practical implications. *Antioxid. Redox Signal.* **11**(4), 861-905. (doi:10.1089/ars.2008.2177).

8. Dietz KJ, Pfannschmidt T. 2011 Novel Regulators in Photosynthetic Redox Control of Plant Metabolism and Gene Expression. *Plant Physiol.* **155**(4), 1477-1485. (doi:10.1104/pp.110.170043).

9. Foyer CH, Bloom AJ, Queval G, Noctor G. 2009 Photorespiratory Metabolism: Genes, Mutants, Energetics, and Redox Signaling. In *Annual Review of Plant Biology* (pp. 455-484. Palo Alto, Annual Reviews.

10. Kutschera U, Niklas KJ. 2013 Metabolic scaling theory in plant biology and the three oxygen paradoxa of aerobic life. *Theory in Biosci.* **132**(4), 277-288. (doi:10.1007/s12064-013-0194-3).

11. Speijer D. 2017 Alternating terminal electron-acceptors at the basis of symbiogenesis: How oxygen ignited eukaryotic evolution. *Bioessays*, 1600174. (doi:10.1002/bies.201600174).

12. Roldan-Arjona T, Ariza RR. 2009 Repair and tolerance of oxidative DNA damage in plants. *Mutat. Res.-Rev. Mutat. Res.* **681**(2-3), 169-179. (doi:10.1016/j.mrrev.2008.07.003).

13. Allen JF. 2015 Why chloroplasts and mitochondria retain their own genomes and genetic systems: Colocation for redox regulation of gene expression. *Proc. Natl. Acad. Sci.* **112**(33), 10231-10238. (doi:10.1073/pnas.1500012112).
