# Supplementary material for: How oxygen gave rise to eukaryotic sex
Source: Proc Biol Sci. 2018 Feb 7;285(1872):20172706. doi: 10.1098/rspb.2017.2706 (PMC5829205; doi:10.1098/rspb.2017.2706)
Supplement: The interplay of multicellularity, meiotic sex and ROS [file rspb20172706supp3.docx]

**Supplementary electronic material S3:**

**The interplay of multicellularity, meiotic sex and ROS.**

Multicellularity arising from single cell stages was coupled to meiotic sex from the start. Otherwise multiple meiosis-mixis cycles in a syncytium would result in nuclei with different gene combinations, and hence in competition of their varying transcription products. Of note, a syncytial pre-eukaryote might have been a genetic experimental phase during the establishment of eukaryotic metabolism [1], but that phase, if it ever occurred, was over by the time that full-blown meiotic sex was established in LECA, let alone when multicellular eukaryotes started to appear. A coordinated developmental program for complex multicellularity can arise only from nuclei that have an identical genome that is multiplied by many mitoses. Since all daughter cells of the initial cell share the same genome, possible conflict and competition among sister cells is minimized [2].

From the perspective of sex as a tool for DNA repair, it is most parsimonious if the zygote, the product after cell fusion, has thoroughly repaired nuclear DNA, as it will carry the blueprint for all nuclear DNA in the daughter cells of the organism [3]. Again, to perform HR repair for all nuclei of a syncytium would be quite costly and inefficient. Hence, meiosis and mixis are combined before the mitotic cell division, differentiation, and growth phase of the organism would start. New gene combinations arising from the tightly controlled recombination events would thus be inherited by all daughter cells. These were also selective forces for coupling sex to reproduction [3].

ROS do not only have negative effects, but play a role in functional cell differentiation by influencing posttranslational protein modification, transcription and gene expression [4] and also in programmed cell death [5], mechanisms all essential for the differentiation and maintenance of more complex tissues [6, 7]. These positive effects are nicely reflected in the concept of mitohormesis, stating that ROS may also function as signalling molecules delaying chronic diseases and even lengthening lifespan in animals [8-10]. Complex multicellularity allowed tissue differentiation with different levels of ROS production in different cell types with widely differing metabolic phenotypes; see also [11] and references therein. The separation of germline cells represents the most decisive cell differentiation step. The germ-soma differentiation allows an efficient reduction of the costs of sex, while also increasing the chances of survival [12]. Other than in protists, meiosis can now run in germline precursor cells in parallel with activities of somatic cells, with minimized metabolic oxidative stress, and without the lethal risk of failure; even if some meiotic divisions fail, the multicellular organism just produces another set of gametes. All other cells became the somatic line to differentiate for other survival enhancing functions.

Non-dividing somatic cells retain only the many non-HR DNA repair mechanisms which are more prone to give rise to base alterations [13, 14]. HR repair is also still possible during mitosis in somatic cells when sister chromatids are available as templates [13]. However, sister chromatids are more likely to have experienced similar damage and are probably less efficient templates for HR repair than meiotic homologous chromosomes derived from different parents [15]. Moreover, in post-mitotic tissues the mitotic HR repair tool becomes unavailable. This might strongly affect neuronal cells as well as cells in other somatic tissues. Strikingly, many metazoans start with reproduction when they stop growing; also angiosperms develop flowers on shoots that have stopped growth. The consequence for somatic cells is a long-term accumulation of oxidative damage on the one hand, and accumulation of base alterations resulting from incorrect non-HR repair mechanisms on the other. Accumulation of oxidative damage on all cellular components (nucleic acids, lipids, proteins), especially in mitochondria, makes eukaryotic somatic cells vulnerable to ageing and finally, destined to die [16]. But, the selective advantages of having high energy metabolisms (oxidative respiration and water-dependent photosynthesis) combined with multicellular tissue differentiation outweigh the price of somatic death. In the true sense of the word, eukaryotes die for energy.

**References**

1. Garg SG, Martin WF. 2016 Mitochondria, the cell cycle, and the origin of sex via a syncytial eukaryote common ancestor. *Genome Biol. Evol.* **8**(6), 1950-1970. (doi:10.1093/gbe/evw136).

2. Rokas A. 2008 The molecular origins of multicellular transitions. *Curr. Opin. Genet. Dev.* **18**(6), 472-478. (doi:10.1016/j.gde.2008.09.004).

3. Hörandl E. 2009 A combinational theory for maintenance of sex. *Heredity* **103**(6), 445-457. (doi:10.1038/hdy.2009.85).

4. Allen JF. 2015 Why chloroplasts and mitochondria retain their own genomes and genetic systems: Colocation for redox regulation of gene expression. *Proc. Natl. Acad. Sci.* **112**(33), 10231-10238. (doi:10.1073/pnas.1500012112).

5. Mittler R. 2017 ROS Are Good. *Trends Plant Sci.* **22**(1), 11-19. (doi:<http://dx.doi.org/10.1016/j.tplants.2016.08.002>).

6. Moller IM, Jensen PE, Hansson A. 2007 Oxidative modifications to cellular components in plants. In *Annual Review of Plant Biology* (pp. 459-481. Palo Alto, Annual Reviews.

7. Holmstroem KM, Finkel T. 2014 Cellular mechanisms and physiological consequences of redox-dependent signalling. *Nat. Rev. Mol. Cell Biol.* **15**(6), 411-421. (doi:10.1038/nrm3801).

8. Ristow M, Schmeisser K. 2014 Mitohormesis: promoting health and lifespan by increased levels of reactive oxygen species (ROS). *Dose-Response* **12**(2), 288-341. (doi:10.2203/dose-response.13-035.Ristow).

9. Held NM, Houtkooper RH. 2015 Mitochondrial quality control pathways as determinants of metabolic health. *Bioessays* **37**(8), 867-876. (doi:10.1002/bies.201500013).

10. Yun J, Finkel T. 2014 Mitohormesis. *Cell Metabolism* **19**(5), 757-766. (doi:10.1016/j.cmet.2014.01.011).

11. Speijer D. 2016 Being right on Q: shaping eukaryotic evolution. *Biochem. J.* **473**, 4103-4127. (doi:10.1042/bcj20160647).

12. Michod RE. 2007 Evolution of individuality during the transition from unicellular to multicellular life. *Proc. Natl. Acad. Sci.* **104**, 8613-8618. (doi:10.1073/pnas.0701489104).

13. Bleuyard JY, Gallego ME, White CI. 2006 Recent advances in understanding of the DNA double-strand break repair machinery of plants. *DNA Repair* **5**(1), 1-12. (doi:10.1016/j.dnarep.2005.08.017).

14. Friedberg ECW, G.C.; Siede, W.; Wood, R.D.; Schultz, R.A.; Ellenberger, T. 2006 *DNA Repair and Mutagenesis*. 2 ed. Washington D.C., American Society for Microbiology.

15. Hörandl E, Hadacek F. 2013 The oxidative damage initiation hypothesis for meiosis. *Plant Repr.*  **26**(4), 351-367. (doi:10.1007/s00497-013-0234-7).

16. Allen JF. 1996 Separate sexes and the mitochondrial theory of ageing. *J. Theor. Biol.* **180**(2), 135-140. (doi:10.1006/jtbi.1996.0089).
